# Design and Validation of a Virtual Physical Education and Sport Science–Related Course: A Learner’s Engagement Approach

**DOI:** 10.3390/ijerph19137636

**Published:** 2022-06-22

**Authors:** Vijayamurugan Eswaramoorthi, Garry Kuan, Mohamad Razali Abdullah, Anwar P. P. Abdul Majeed, Pathmanathan K. Suppiah, Rabiu Muazu Musa

**Affiliations:** 1 Faculty of Health Science, School of Rehabilitation Science, Universiti Sultan Zainal Abidin, Gong Badak Campus, Kuala Terengganu 21300, Terengganu, Malaysia; vijayeswar@unisza.edu.my; 2 Exercise and Sports Science Programme, School of Health Sciences, Universiti Sains Malaysia, Kubang Kerian 16150, Kelantan, Malaysia; 3 East Coast Environmental Research Institute, Universiti Sultan Zainal Abidin, Kuala Terengganu 21300, Terengganu, Malaysia; razaliabdullah@unisza.edu.my; 4 Innovative Manufacturing, Mechatronics and Sports Laboratory, Faculty of Manufacturing Engineering, Universiti Malaysia Pahang, Pekan 26600, Pahang, Malaysia; amajeed@ump.edu.my; 5 School of Robotics, XJTLU Entrepreneur College (Taicang), Xi’an Jiaotong-Liverpool University, Suzhou 215123, China; 6 Faculty of Psychology and Education, Universiti Malaysia Sabah, Kota Kinabalu 88450, Sabah, Malaysia; pathmaha@ums.edu.my; 7 Centre for Fundamental and Continuing Education, Universiti Malaysia Terengganu, Kuala Terengganu 21030, Terengganu, Malaysia

**Keywords:** health promotion, virtual teaching and learning, learner’s engagement, course design, online physical education–sport science course, out-of-class engagement

## Abstract

Learners’ engagement is shown to be a major predictor of learning, performance, and course completion as well as course satisfaction. It is easier to engage learners in a face-to-face teaching and learning format since the teacher can observe and interpret the learner’s facial expression and body language. However, in a virtual setting with the students sitting behind cameras, it is difficult to ascertain engagement as the students might be absent-mindedly attending the class. Henceforth, with the rapid transition to online learning, designing course content that could actively engage the students towards achieving the said elements is, therefore, necessary. We applied a data-driven approach in designing a virtual physical education and sport science–related course via a learner engagement model. A fully online course catering to 132 students that runs for a total of 14 weeks was used as a case study to develop the course. The study was conducted during the 2020/2021 academic year, which was the period of the peak COVID-19 pandemic in Malaysia. The delivery of the course content was implemented in stages to achieve three essential educational outcomes namely, skill and knowledge acquisition, and personal development as well as course satisfaction. We hypothesised that the developed learners’ engagement approach will promote the students’ acquisition of skills and knowledge and foster the personal development of the students through fitness improvement. It is also hypothesised that the students will be satisfied with the course developed upon successful completion. A chi-square analysis projected a statistically significant difference in the skill and knowledge acquisition before and after the programme (*p* < 0.001). A Wilcoxon rank-sum test demonstrated personal improvement in the overall fitness of the student upon completing the prescribed activity of the course content. Moreover, a total of 96.2%, 95.5% and 93.2% of students expressed their satisfaction with the clarity of the learning objectives, good organisational and course content plan, and appropriate workload of the course designed, respectively. There is sufficient evidence to accept all hypotheses formulated, and hence, we postulated that, since students spend more time outside the classroom, out-of-class learners’ engagement activity should be considered when designing a virtual course to promote lifelong learning, experience, and higher-order thinking. The techniques presented herein could be useful to academics, professionals, and other relevant stakeholders in developing virtual course content within a specific domain of interest.

## 1. Introduction

For at least a century, the teaching and learning process has been undertaken in a face-to-face mode; however, due to the advancement in science and technology and pedagogy as well as the increasing need for working adults to acquire and upgrade their knowledge as lifelong learners, the gradual transformation from a conventional learning mode to digital learning programmes ranging from blended (hybrid) to fully online virtual classes are being witnessed. Students’ interest in online courses and programmes is now being recognised by colleges and universities. The student’s preference towards virtual online educational opportunities is reported to be associated with increased access, flexibility, convenience, technological and format ubiquity, and cost [1]. Conversely, in a competitive higher education market, virtual online courses also promise benefits to institutions, such as increased reach and sustainability [2]. For instance, it was reported that in 2016, about 31.6 percent of students enrolled in at least one online education course, and the trend towards growth in online enrolments has progressively increased despite a decline in overall higher education enrolments [3]. The challenge thus far is, How to effectively deliver online courses?

It is rather inappropriate and a mistake to assume that the delivery of an effective online course involves simply shifting classroom content, assignments, and exams to a Web-based setting. It is worth noting that all institutions of learning are dedicated to ensuring the delivery of a learning experience that could promote educational depth, critical thinking and active learning engagements irrespective of the format of the course. Essentially, any element of teaching and learning is expected to equip students with exposure to instructional materials, learning activities and in-class and out-of-class interactions to enable the acquisition of information, skill, and knowledge aimed at activating a higher order of thinking towards solving a real-life problem through active learning engagement [4,5]. Nonetheless, many academics are still pondering the kind of activities that could promote and provide learning engagement for online students.

Previously, researchers have inferred that learning engagement is constituted of physical and mental energy, is situation specific, and is a blend of both quantitative and qualitative elements [6]. In a later investigation, students’ engagement was shown to be substantially strengthened via academic participation, involvement with an institution, and student peer groups amongst college students [7,8]. This evidence highlights that the social measures of learning engagement involve three major elements, namely, behaviours, emotions, and cognitive engagement, in both conventional and online learning [9,10,11]. Behavioural engagement typically involves students partaking in activities related to the course projects or assignments [12]. The emotional engagement reflects the student’s attitude with respect to the teachers, their fellow students, or the course entirely [13]. Conversely, cognitive engagement refers to the student’s evaluation of the course content as both pertinent and valuable [14]. This segmentation of learning engagement elements has given rise to a better understanding of students’ engagement as it relates to the classroom activities, and thus, researchers began developing a model for the concept.

A community of inquiry (CoI) model–based learning engagement was developed to decipher what transpires in online virtual courses with regard to interactions [15]. The model illustrated that effective engagement involves three manifestations: i.e., the social, teaching, and cognitive presences [16,17]. The social presence revolves around the ability of students to view their characteristics and make themselves presentable with the sole purpose of maintaining relations with their peers. The teaching presence is seen as the development and facilitation of the cognitive as well as social processes in such a way that the students could personally acquire meaningful educational outcomes. The cognitive presence, on the other hand, is the degree to which the students are capable of establishing meaning in continuous communication [15,18]. It is important to note these three presences are not only correlated to each other but also associated with students’ engagement. Evidence demonstrated that about 70 percent of the variance in the cognitive presence of online students’ scores is explained by the perceived levels of both teaching and social presence [19]. Similarly, studies conducted across 30 virtual online classes revealed a positive significant relationship between students’ perceptions of a sense of community and learner engagement [17].

The difference between conventional teaching and learning and virtual learning appeared to be centred around learner engagement. For instance, in conventional classes, the teacher could evaluate the facial expressions of the students to determine the existence of any ambiguity as well as to ascertain whether the concept being introduced is understood or not, without necessarily asking questions. This could allow the teacher to re-explain or demonstrate the concept to clear any misunderstanding or ambiguity. On the other hand, the aforesaid opportunity may not transpire in a virtual class due to the nature of the learning involved in online classes. In a virtual class, the students sit behind their laptops/computer screens, which makes it difficult to measure learning engagement via facial expression or body language. Some students might be physically present behind their cameras, but they could be engrossed in other activities such as online games, checking emails, chatting through social networks, or even moving away from the class entirely [20]. It is important to note that learning engagement may not necessarily account for students’ overall comprehension as other influential factors may exist; however, it plays a vital role in supporting and improving students’ understanding.

It was suggested by a previous study that physical-education-related programmes in schools should focus on curricular activities directed towards the promotion of lifelong well-being [21]. Moreover, it has been reported that the second decade of life is deemed the most critical period in which behavioural health is shaped. Hence, intervention programmes are mostly conducted at this age in both schools and family settings [22]. However, it is worth highlighting that the school intervention programmes are shown to be vital as they have the capacity to reach larger populations, thereby reducing social inequalities in health [23]. It is, therefore, imperative to design a virtual course that could address the problem of poor learning engagement during virtual classes, particularly for the physical education and sport science–related course, which is primarily psychomotor based because of the course’s emphasis on the students’ acquisition of movement skills and its application in daily life.

### Conceptual Framework

The emergence of COVID-19 and the intermittent spikes in positive cases have resulted in the conversion of regular classes to online or virtual classes. The term online and virtual classes are often used interchangeably. However, the two terms differ. The terms online learning and virtual learning are unique in characteristics; although each reflects the act of applying technology in teaching and learning, how participants interact in the process and engage with the learning materials is different. In online learning, students learn at their own pace and convenience without the assistance of a teacher or an instructor. All learning materials (videos, notes, slides, lectures, tests quizzes, homework, and assignments) are made easily available for the students who enrolled in the course [24]. On the other hand, virtual classes are normally arranged in a live session mode with a teacher or an instructor guiding all the processes remotely [25]. In the present study, we endeavour to blend the two formats of learning, i.e., online and virtual, due to the nature of sports science and physical education–related courses in which the students are required to master and exhibit some of the complex movements correctly. Hence, creating the need to provide a virtual guide on the execution of such movements.

**Figure 1:**
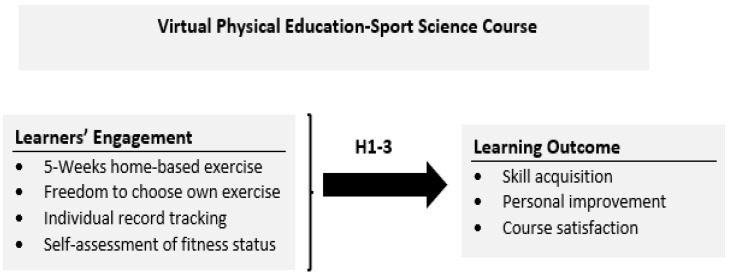
Conceptual framework of the study.

It has been demonstrated from the previous framework that learners’ engagement is a significant predictor of learning performance, course completion, and course satisfaction in online or virtual courses [15]. Moreover, students are found to be motivated and actively engaged in online or virtual classes when they are given the autonomy to choose and determine the pace of learning independently via guided discovery [26,27]. When students are fully engaged in a course, positive learning outcomes comprising skill and knowledge acquisition, as well as satisfaction, are likely to be achieved [28,29]. In this regard, we developed the current framework shown in Figure 1 to design a virtual physical education and sport science–related course that could provide exposure for students through instructional materials, learning activities and interactions geared towards the acquisition of information, skill, and lifelong learning experience. We seek to achieve these objectives through a case study of a fully online virtual class. The study herein specifically focused on achieving three essential educational outcomes namely, skill and knowledge acquisition, personal development, and course satisfaction. The activities itemised in Figure 1 (5 weeks home-based exercise, freedom to choose own exercise, individual record tracking, and self-assessment of fitness status) are termed as “learning engagement” because the activities fully revolved around the students without the teacher’s intervention. Thus, the study seeks to validate the framework through the collection of relevant data since analysis and interpretation are crucial for the successful attainment of any course design. In addition, it is inferred that the process of interpreting outcomes in an intervention programme needs to consider certain methodological standards [30]. Firstly, an inference of evidence-based efficacy in the study should be provided. Secondly, the criterion of evaluation should be clearly stated, and finally, the interactions of the contextual variables in the course implementation should be taken into account. Therefore, in line with the said elements, the following hypotheses are formulated to guide the conduct of the study:

**H1.**  *The learners’ engagement approach developed will promote the students’ acquisition of skills and knowledge.*

**H2.**  *The learner’s engagement approach utilised in the study could foster the personal development of the students through fitness improvement.*

**H3.**  *The students will be satisfied with the course developed upon successful completion.*

The dependent variable for the first hypothesis is the perceived acquisition of skill and knowledge whereas the independent variables are the ratings of the skill and knowledge ranging from a score of 1 (poor) to 5 (excellent). The dependent variable for the second hypothesis is the personal improvement of the students with respect to fitness status while the independent variables are the flexibility and a combination of core and upper muscle endurance. Similarly, the dependent variable for the third hypothesis reflected the perceived satisfaction of the students upon completing the course whilst the independent variables constitute the ratings of the students in the three essential elements of the course content, viz., clear learning objectives, good organisation, and course content plan as well as appropriate workload rated from a scale of 1 (strongly disagree) to 5 (strongly agree).

## 2. Materials and Methods

### 2.1. Participants

The participants of the present study comprised a total of 132 undergraduate students (43 males and 89 females), with the following characteristics: body mass index (23.38 ± 13.31) and age (20.02 ± 0.96), mean and standard deviation, respectively. The participants were undergraduate students undertaking a physical education and sports science–related subject in one of the public universities in Malaysia. Like all other physical education and sports science–related courses, the students were expected to acquire knowledge and skills regarding physical exercises, the nature of various sports and physical activity as well as ways in which the students could live a healthy lifestyle through balanced nutrition and physical activity. It is worth mentioning that, before the commencement of data collection in this study, the standard guidelines for research involving human subjects as recommended by the International Declaration of Helsinki were observed [31]. While all the students were expected to participate fully in the physical activity involved in the course, care was taken to ensure that the students were healthy and fit to undergo the physical activity. Therefore, health screening was carried out via an online survey to determine the presence of any ailment or physical deformity that could interfere with the student’s engagement in physical activity. In the event that the student reported any concern that student was given a special recommendation and unique activity that was safe to perform. It is also important to note that after approval was obtained to carry out the study, all the participants signed a constant form.

### 2.2. Course Implementation and Data Collection Procedure

**Figure 2:**
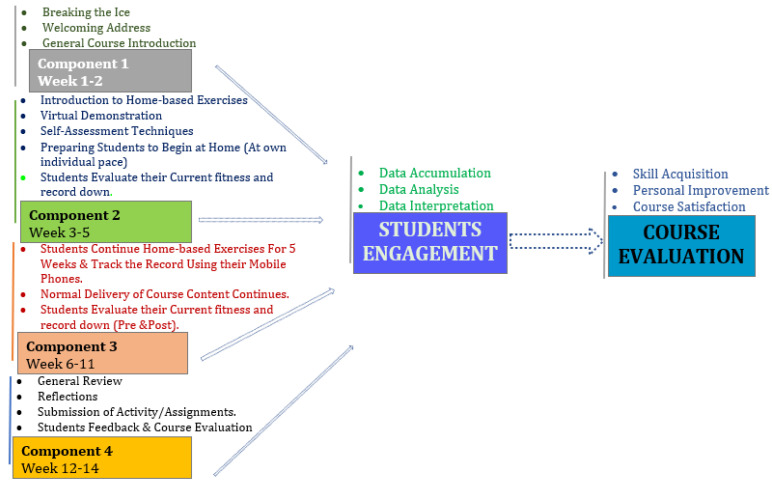
Methodological flowchart of the course design.

The design of the current virtual course was carried out in phases as depicted in Figure 2. These phases were deemed necessary in order to achieve the objective of the study of designing a reliable and valid online physical education and sports science–related course. As noted in the previous studies highlighted above, to design any online course, the course content needs to capture the acquisition of knowledge and skills, the personal development of the students, and the course satisfaction derived by the students upon the successful completion of all the course content. Therefore, considering these important elements, we developed the phases highlighted in Figure 2. After the first two weeks of the introductory stage of the course, the participants were guided on the testing and measurement of the basic anthropometric variables, i.e., weight and height, whilst the students were also required to document their personal information such as age, gender, and race. Furthermore, the fitness-related attributes, which included flexibility and upper and core muscle strength, were demonstrated to the students step by step by the course lecturer who was an experienced physical education and sport science teacher.

After the students were familiar with all the testing and measurements such that each student could independently carry out the assessment, the students were asked to conduct a pre-testing on their overall physical fitness status to determine their present level of fitness. The students were then required to download any application on their mobile phones that could track their daily physical activity before the commencement of the exercises. The students were permitted to choose any home-based physical activity in accordance with their level of fitness and carry out the exercise for 5 weeks and at least 3 days per week. The students were permitted to choose any exercise they preferred in order to capture different levels of fitness and to give room for students with both low- and high-fitness status to participate in the course. This is considered vital since students possess different characteristics in a typical class (heterogeneous).

The data that contain information such as daily steps per day, the type of home-based exercise performed, the duration of the exercise, and the screenshots of the exercise summary were collected from the students at the end of every week. The students were familiarised with all the processes prior to the full commencement of the data collection. It is worth noting that all the processes were carried out virtually such that no physical contact transpired during the data collection process.

### 2.3. Measures of Success upon Course Completion

In the current study, skill acquisition, personal improvement, and course satisfaction were considered as measures for success upon completion of the prescribed course content. These elements were selected as the basis for evaluating the students’ achievement as teaching is a pedagogical process that involves the technique of imparting skill and knowledge to the students, facilitating personal development through instilling lifelong learning ability as well as inducing student satisfaction [32,33].

The acquisition of skill and knowledge is fundamental to the teaching and learning processes. The teaching and learning materials should be designed in such a way that active learning methodologies are put in place to permit a high level of student involvement, which is essential in fostering dynamic learning and greater interactions with the contents [34]. This can be achieved when students are guided through the organisation of activities that are not only completed in the classroom but also extended outside the classroom [35]. To this end, the student’s achievement in the acquisition of skill and knowledge is determined by comparing the level of skill and knowledge of the students before and after the attainment of the course. This technique is shown to be effective for evaluating students’ achievements in various fields [36,37]. Moreover, it is inferred that personal growth and self-improvement are reported as essential attributes that could be acquired in educational settings where the acquisition of knowledge and skill culminates into the attainment of desirable outcomes [38]. In this regard, the skill imparted to the students could be used to improve practice in a professional context, thereby, creating behavioural changes resulting in positive impact [39,40]. In this study, the fitness status of the students with respect to the differences before and after instruction in the fitness parameters, i.e., flexibility and a combination of core and upper muscle endurance, were investigated.

Satisfaction coupled with positive feelings concerning teaching methodology has been highlighted as the key ingredient for the attainment of success in different modes of course design [41]. Hence, it is vital to integrate a pedagogical model in which the learning methods are implemented based on the learner’s interests and needs [42]. Virtual learning has the capacity to offer significant advantages over conventional classroom learning in the aspect of accessibility, ease, and convenience [43]. However, it requires careful consideration of the essential factors of learning to promote and sustain students’ interest. In this regard, we applied the notion put forward in the ARCS model (attention, relevance, confidence, and satisfaction) by a preceding researcher [44,45]. The model emphasised that a lesson must be able to gain and sustain the attention of learners. This can be achieved through the introduction of varying activities to stimulate a sense of enquiry in the learners. Secondly, there is a need to establish relevance in the lesson. Here, learners must perceive the instructional materials as relevant and consistent with a set goal. Thirdly, confidence encapsulates helping students to have positive expectancies for success based on their peculiar abilities. This can be achieved via the allocation of an appropriate workload within the course materials. Considering the aforementioned elements, we assessed the student’s satisfaction based on the three essential elements, namely, clear learning objectives, good organisation and course content plan, and appropriate workload.

### 2.4. Data Analysis

The normality of the data was verified using Shapiro–Wilks. It was observed that the data did not follow a normal distribution, thus, the application of non-parametric tests for analysis and interpretation of the data gathered were deemed applicable in the present investigation. The Wilcoxon rank-sum test was used to measure the personal improvement of the students as lifelong learners; hence, the effectiveness of the intervention (five weeks based home-based exercises) on the fitness status of the students with respect to the differences before and after instruction in the evaluated fitness parameters, i.e., flexibility and a combination of core and upper muscle endurance, were investigated. The Wilcoxon rank-sum test is considered appropriate in this study due to its ability to cater for non-normal data distribution as suggested by the preceding researchers since the participants were evaluated in two different times (before and after) [46,47]. Moreover, it is important to highlight that a reliability analysis was carried out to establish the authenticity of the measurements in evaluating the health and the fitness parameters of the participants since all the measurements were conducted virtually. The reliability analysis was carried out by correlating the pre-test and post-test values of all measured variables. A Cronbach alpha value of 0.719 was observed, indicating the internal reliability and consistency for both the pre-test and post-test values of the measured variables. Additionally, correlation coefficients ranging from 0.88 to 0.98 were found, further revealing the accuracy of the measurements across all variables.

Moreover, to examine skill and knowledge acquisition coupled with the course satisfaction derived by the students upon completing the course, a questionnaire was developed to measure whether the students were able to acquire the skills and knowledge upon attending the course with the questions ranging from poor (score 1) to excellent (score 5). The other construct of the questionnaire also included a measure of their course satisfaction with respect to the course content: Are the learning objectives clear? Is the course content organised and well planned? Is the workload appropriate? These questions ranged from strongly disagree (scale 1) to strongly agree (scale 5). Reliability analysis was also carried out to determine the items’ reliability as well as the consistency of the responses. An overall Cronbach alpha value of 0.945 was detected, revealing the internal reliability and the consistency of the items. Strong correlation coefficients of 0.885, 0.891, and 0.884 for clear learning objectives, organisation of the course content, and appropriate workload were also observed, respectively. It is worth noting that the students answered the questionnaire at the end of the course. A chi-square analysis was employed to analyse the responses gathered from the questionnaire survey. The statistical analysis was carried out utilising XLSTAT 2014 Addinsoft Inc., New York, NY, USA, add-in software for Windows and SPSS ver.25 for Windows. All the inferences were set at *p* ≤ 0.05.

## 3. Results

**H1.**  *The learners’ engagement approach developed will promote the students’ acquisition of skills and knowledge*.

Table 1 shows the comparison of the degree of knowledge and skills at the start of the course as well as at the end of the course. A statistically significant difference was observed in the students’ levels of skills and knowledge before and after the course *p* < 0.0001. It can be seen from the table that a total of 58.3%, 31.8%, 8.3%, and 1.5% of the students recorded excellent, very good, satisfactory, and fair skill and knowledge acquisition at the end of the course, respectively. Whilst a total of 34.1%, 23.5%, 21.2%, 18.2%, and 3% were within the level of excellent, very good, satisfactory, fair, and poor skill and knowledge categories before attending the course. This evidence demonstrates that the course content was effective in providing the students with the necessary learning materials and experiences to enhance their knowledge and skills within the subject matter. Moreover, no statistically significant difference was observed with respect to gender in the acquisition of skills and knowledge before and after attending the course, *p* = 0.234; 0.944, respectively.

**Figure 3:**
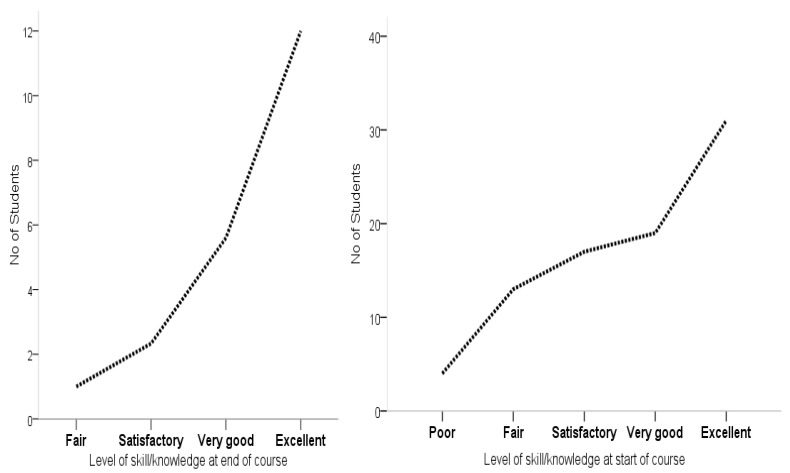
Levels of skill and knowledge before and after the course.

Figure 3 depicts the levels of skill and knowledge of the students before and after attending the course. It could be observed that a higher number of students fell within the categories of very good and excellent skill and knowledge acquisition after the programme as opposed to before attending the course when the students were dispersed into poor, fair and satisfactory categories. It is worth highlighting that no student was found to be within the poor category of knowledge and skill upon course completion. This echoed the relevance of the materials towards imparting the necessary skill and knowledge to the students.

**H2.**  *The learner’s engagement approach utilised in the study could foster the personal development of the students through fitness improvement.*

**Figure 4:**
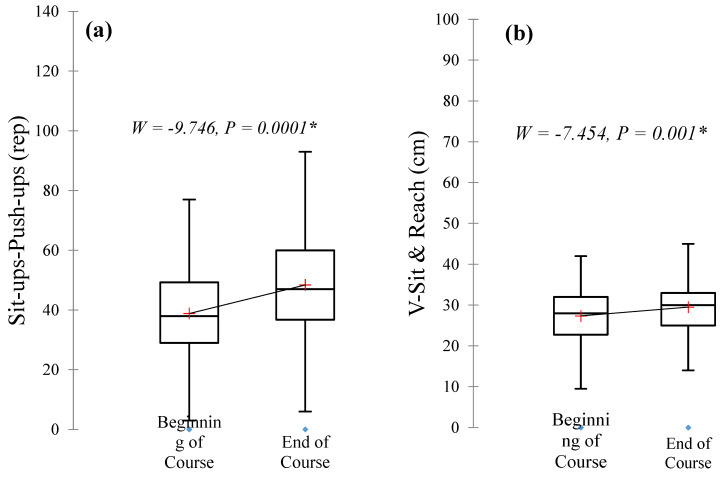
Muscle functionality differences before and after the course. (**a**) Core muscle and upper muscle endurance and (**b**) flexibility. * Wilcoxon signed rank test.

Table 2 tabulates the effectiveness of the 5 weeks of physical activity on the fitness and health parameters of the students. The 50th, 25th, and 75th percentile of the medium scores, and the descriptive and inferential statistics of the variables investigated are shown in the table. It can be observed that the mean values of both the muscle flexibility and fitness measures increased within the activity period. Interestingly significant differences were detected in the flexibility and upper and core muscle strength of the participants after the intervention period. This demonstrated that a certain level of personal development was achieved by the students as a result of attending and completing the course programme. Moreover, Figure 4 depicts the effectiveness of the programme in improving the muscle functionality of the students.

Figure 3 portrays the mean performance difference plots of the significant variables with respect to the pre- and post-physical activity level of the student examined in the course. It could be observed from the boxplots that the said parameters, i.e., flexibility, core muscle endurance and upper muscle endurance of the students, were higher after completing the course as compared to before starting the course. This signifies that the students acquired the skill and knowledge that could guide them towards achieving better health through the course. It is also an indicator that the course design was effective in encouraging the students to continue and sustain the activity even after the class, as many students reported their commitment to continuing the activity even after the 5 weeks.

**H3**  . *The students will be satisfied with the course developed upon successful completion.*

Table 3 reveals the students’ perception of the satisfaction derived after attending the course. The students’ responses based on the three main elements, i.e., clear learning objectives, good organisational and course content plan, and appropriate workload, are tabulated. It could be seen that a total of 71.2 percent strongly agreed, and 25 percent of the students agreed that the course has clear learning objectives whilst only 2.3 and 1.5 percent were neutral and strongly disagreed, respectively. On the good organisation and course content plan, a total of 75.8 percent strongly agreed, and 19.7 percent of the students recorded their agreement that the course was well organised and planned, whilst only 3 and 1.5 percent recorded neutral and strongly disagreed, respectively. Likewise, a total of 69.7 percent strongly agreed and 23.5 percent of the students agreed that the workload within the course was appropriate, while 5.3 and 1.5 percent remained neutral and strongly disagreed, respectively. Generally, it could be deduced that a higher percentage of the students positively agreed that significant satisfaction was derived owing to the attendance of the course. It is worth noting that no statistical differences were observed based on the gender of the students in their perceived rating of satisfaction upon attending the course.

## 4. Discussion

The current study is aimed at designing a virtual physical education and sports science–related course that could provide exposure for students to instructional materials, learning activities, and interactions geared towards the acquisition of information, skill, and learning experience. Bearing in mind that any effective virtual online programme should be able to adequately impart skill and knowledge to the students, facilitate personal development via instilling lifelong learning ability, and evoke satisfaction in the students, we objectively employed a case study technique for a fully online course that lasted for 14 weeks to design, validate, and measure the satisfaction of the students. It is worth highlighting that, to the best of our knowledge, the current investigation is the first attempt at designing a physical education and sport science–related virtual course. Owing to the importance of the data-driven approach in proving reliable information about a given phenomenon, we developed and validated the course through the collection of relevant data in a case-study format since analysis and interpretation are crucial towards the successful attainment of any course design.

It was demonstrated from the findings of the current study that the course design is effective in imparting skills and knowledge to the students upon the completion of the programme as evident in Table 1 and Figure 2. This achievement is determined as a result of the knowledge and skill evaluation by comparing the level of skill and knowledge of the students before and after the programme. This finding supported our hypothesis that the learner’s engagement approach utilised could promote the student’s acquisition of skill and knowledge. Evaluation of the skill and knowledge of the student in online learning is seen as a technique undertaken by academics and professionals in every discipline via a comparison of the initial level of skill and knowledge with the predicted or the actual skill acquired. In this regard, the suitability, as well as the efficacy of the course, is determined and, hence, provides a guide for action [5]. Moreover, as the virtual learning method is rapidly gaining recognition as an acceptable way of providing knowledge and training, a priority should then be set to ensure that the knowledge and skill obtain in the classroom during the conventional teaching and learning process is seamlessly transferred across into the virtual learning settings [48,49]. Online learning, as facilitated by technology, enables the dissemination of course content to several learners, which provides opportunities for the learners to have control over the content as well as the time spent on learning the content. This process could be modified to suit the needs of the learners and the course learning outcomes [50]. This could contribute towards providing a better learning experience that is non-trivial in enhancing the students’ learning process [51,52].

It was hypothesised (H2) that the learner’s engagement approach utilised in the study could foster the personal development of the students through fitness improvement. The findings from the current investigation also revealed that the students were able to develop and enhance their fitness statuses upon completing the programme as depicted in Table 2 and Figure 3. Essentially, the muscle flexibility of the students increased significantly. Flexibility is an important part of physical fitness and has many health benefits. For example, it increases agility, balance, and muscle control and lowers the risk of injury and muscle soreness. It also results in a better overall “form.” It mostly expands the range of motion and makes the exercises easier to do. The current finding is in agreement with a previous study that demonstrated that flexibility could be achieved by a variety of methods that may not necessarily be structured, ranging from bending and foam rolling to regular workouts [53,54,55]. Failure of an individual to regularly train for flexibility could result in a gradual shortening of muscles. For instance, the hip flexor muscle might lose its elasticity due to long hours of sitting [56]. It is important to highlight that limited flexibility often results in stiffness that restricts the smooth conduct of daily activity as well as limiting exercise dexterity.

The other health-related markers that were significantly improved between the pre- and post-activity periods of the course were 1-min sit-ups and 1-min push-ups. These two tests were used to determine the muscular endurance of the participants. A previous study found that physical activity was inversely associated with muscle fatigue [57]. This signifies that people who always participated in physical activity normally have better muscle endurance. Moreover, many studies have shown that muscular endurance improved after several days of exercise or training [58,59]. This adaptation occurred for several reasons. One of them is the improvement of blood circulation in the muscles. Good blood circulation will result in a good buffer system. For example, when an individual is involved in vigorous or high-intensity exercise, the body will produce a high concentration of lactic acid in the blood. This lactic acid can impair muscle contraction and result in muscle tiredness and cramps. However, for those who have good blood circulation, the lactic acid could easily be transported to the liver and converted to glucose. This will reduce the lactic acid accumulation in the muscle and prevent muscle cramps, leading to muscle endurance in an individual.

The satisfaction derived by the students as a result of attending the course is apparent in Table 3. The satisfaction of the students was determined based on the three essential elements for any teaching and learning activity. The students showed their satisfaction with the clear learning objectives, good organisational and course content plan, and appropriate workload. Despite the enormous advantages of online learning some challenges exist, such as student retention and loss of interest, and the dropout rate is substantially higher compared to that in conventional classroom teaching and learning [60,61]. These challenges elicited questions from many academics about the learning outcomes and sustainability of students’ interests since online learning needs higher self-discipline from the students [62]. While it is noted that online learning does not necessarily require a same-time, same-place mode of education, it may not give room for focused learning especially when the students’ interests and motivation towards learning the skill and knowledge are lacking as a result of the course design [63,64]. Therefore, to mitigate these challenges, evidence has shown that effective pedagogical premises should be considered in the course design. As such, clear learning objectives, good hierarchical organisation of the course content, and an appropriate workload, are essential elements that could withhold the students’ interests and facilitate deeper learning [65].

Our initial hypothesis (H3), which states that the students will drive satisfaction upon completing the course can, therefore, be accepted. However, it is worth noting that the student’s achievement in the course is evident under a short-term i.e., within five weeks of intervention. A previous study carried out under the “*Healthy Me* programme” to assess the effectiveness of the programme in the achievement of physical activity amongst adolescent girls over a period of one year demonstrated no significant changes in the moderate-to-vigorous physical activity of the girls. Only a slight increase in the percentage of the girl’s fulfilling the 7 days of recommended moderate-to-vigorous physical activity was observed [66]. Therefore, whilst it is reported that the use of mobile technology and smart phones applications has the potential to change adolescents’ health-related behaviours [67], the sustainability of such behaviour over a long period remains a challenge. This phenomenon provides an avenue for further research from this perspective.

## 5. Conclusions

In the current investigation, we designed and validated a virtual physical education and sport science–related course. It was demonstrated from the findings of the investigation that the designed course is effective in facilitating the acquisition of skill and knowledge, personal improvement, and satisfaction. Interestingly, besides improving the student’s skills and knowledge, the students were able to enhance their overall physical fitness levels in the aspect of muscular flexibility and endurance, which is pivotal towards maintaining their health and well-being, especially during this period in which the daily business of teaching and learning and other related jobs is carried out at home with little or no physical activity. Moreover, the data-driven approach employed in the current study could be useful to academics, professionals, and other relevant stakeholders in developing course content to suit the requirement of a given course or programme within a specific area of interest.

## 6. Practical Implication

Evolving needs coupled with the COVID-19 pandemic compel the academic community to rethink the notion of educational instructions. The virtual setting is no longer regarded as a temporary necessity but rather as a global disruption in teaching and learning that is here to stay during both the pandemic and beyond. However, the challenge lies in the creation of engaging course content that could offer the learners a better learning experience in tandem with that obtained in conventional face-to-face teaching and learning. A course content that provides exposure for students to instructional materials, learning activities, and interactions to foster the acquisition of information, skill, and knowledge is crucial for online teaching and learning. It is obvious that a “one jacket fits all” course content could not suit the needs of both conventional and virtual teaching and learning. Thus, it is rather inappropriate or wrong to simply transfer a conventional class course content to a virtual class. Hence, the findings from the present investigation could be used as a guideline for teaching virtual physical education and sports sciences–related subjects.

## 7. Limitations of the Study

The present investigation is subject to a few limitations. The autonomy given to the students to choose exercise tracking applications and the possibility of other students completing less or more exercise per week may hinder the unification of each student with others. Moreover, we were unable to recruit a control group to further confirm that the findings were solely caused by the online class. The long-term effects of student achievement could not be ascertained as measurements were only covered before and immediately after the attainment of the course.

## Figures and Tables

**Table 1:** Students’ rating of knowledge and skill acquisition in the course.

Gender	Count (Percent)	Level of Knowledge at Start of the Course					Total
		Poor	Fair	Satisfactory	Very Good	Excellent	
Male	Frequency (%)	0(0)	11(25.6)	10(23.3)	9(20.9)	13(30.2)	43(32.6)
Female	Frequency (%)	4(4.5)	13(14.6)	18(20.2)	22(24.7)	32(36)	89(67.4)
	Overall Total	4(3)	24(18.2)	28(21.2)	31(23.5)	45(34.1)	132(100)
		**Level of Knowledge at End of the Course**					**Total**
		**Poor**	**Fair**	**Satisfactory**	**Very Good**	**Excellent**	
Male	Frequency (%)	0(0)	1(2.3)	4(9.3)	13(30.2)	25(58.1)	43(32.6)
Female	Frequency (%)	0(0)	1(1.1)	7(7.9)	29(32.6)	52(58.4)	89(67.4)
	Overall Total	0(0)	2(1.5)	11(8.3)	42(31.8)	77(58.3)	132(100)
Gender	Starting, χ^2^ (4) = 5.562; *p* = 0.234; ending, χ^2^ (3) = 0.381; *p* = 0.944						
Level of knowledge, before/after		χ^2^ (12) = 79.236; *p* < 0.0001					

**Table 2:** Personal development achieved through the course programme.

Variable	Median (Muscle Functionality)		Z Statistics	*p*-Value *
	Beginning of Class	End of Class		
Flexibility	28 (22, 32)	30 (25, 33)	−7.454	0.001
Muscle fitness	38 (29, 47)	47 (36, 60)	−9.746	0.001

* Wilcoxon signed rank test.

**Table 3:** Students’ perceived rating of course satisfaction derived from the course.

Gender	Count (Percent)	Clear Learning Objectives					Total
		Strongly Disagree	Disagree	Neutral	Agree	Strongly Agree	
Male	Frequency (%)	1 (2.3)	0 (0)	1 (2.3)	13 (30.2)	28 (65.1)	43 (32.6)
Female	Frequency (%)	1 (1.1)	0 (0)	2 (2.2)	20 (22.5)	66 (74.2)	89 (67.4)
	Overall Total	2 (1.5)	0 (0)	3 (2.3)	33 (25.0)	94 (71.2)	132 (100)
		**Good Organisation and Course Content Plan**					**Total**
		**Strongly Disagree**	**Disagree**	**Neutral**	**Agree**	**Strongly Agree**	
Male	Frequency (%)	1 (2.3)	0 (0)	1 (2.3)	10 (23.3)	31 (72.1)	43 (32.6)
Female	Frequency (%)	1 (1.1)	0 (0)	3 (3.4)	16 (18.0)	69 (77.5)	89 (67.4)
	Overall Total	2 (1.5)	0 (0)	4 (3.0)	26 (19.7)	100 (75.8)	132 (100)
		**Appropriate Workload**					**Total**
		**Strongly Disagree**	**Disagree**	**Neutral**	**Agree**	**Strongly Agree**	
Male	Frequency (%)	1 (2.3)	0 (0)	2 (4.7)	13 (30.2)	27 (62.8)	43 (32.6)
Female	Frequency (%)	1 (0.8)	0 (0)	5 (5.6)	18 (20.2)	65 (73.0)	89 (67.4)
	Overall Total	2 (1.5)	0 (0)	7 (5.3)	31 (23.5)	92 (69.7)	132 (100)

Note: No statistical difference across gender.

## Data Availability

The data used are available within the manuscript.
